# Risk factors for *Enterobius vermicularis* infection in children in Gaozhou, Guangdong, China

**DOI:** 10.1186/s40249-015-0058-9

**Published:** 2015-06-02

**Authors:** Hong-Mei Li, Chang-Hai Zhou, Zhi-Shi Li, Zhuo-Hui Deng, Cai-Wen Ruan, Qi-Ming Zhang, Ting-Jun Zhu, Long-Qi Xu, Ying-Dan Chen

**Affiliations:** National Institute of Parasitic Diseases, Chinese Center for Disease Control and Prevention, WHO Collaborative Center for Malaria, Schistosomiasis and Filariasis, Key Laboratory of Parasite and Vector Biology, Ministry of Health, Shanghai, 200025 China; Gaozhou Center for Disease Control and Prevention, Gaozhou, 515200 China; Guangdong Center for Disease Control and Prevention, Guangzhou, 510000 China

**Keywords:** *Enterobius vermicularis*, Prevalence, Risk factor, Children, China

## Abstract

**Background:**

*Enterobius vermicularis* infection is a prevalent intestinal parasitic disease in children. In this study, we explored the epidemiological status and risk factors for *E. vermicularis* infection in children in southern China.

**Methods:**

A cross-sectional survey was carried out in Gaozhou city, Guangdong province, China, in December 2011. Children aged 2–12 years from five schools participated in the study. The adhesive cellophane-tape perianal swab method was applied to detect *E. vermicularis* infection, while a questionnaire was sent to each child’s guardian(s) to collect demographic and socioeconomic data, as well as hygiene behaviors, pertaining to each child. Univariate and multivariate logistic regression analyses were performed to capture the potential risk factors.

**Results:**

Out of the 802 children surveyed, 440 were infected with *E. vermicularis*, with an average prevalence of 54.86 %, and a range from 45.96 to 68.13 %. The age variable was found to be statistically significant, whereas the sex variable was not. It was found that a mother’s education level (low) and not washing hands before dinner were major risk factors in all children (802). After stratification by age, a father’s education level (primary or below) and biting pencils (or toys) were significant risk factors in the younger children (508), while not washing hands before dinner and playing on the ground were important risk factors in the older children (294).

**Conclusion:**

This study demonstrates the prevalence of *E. vermicularis* infection in children in Gaozhou and reveals underlying risk factors. Most importantly, it reveals that risk factors differ among the different age groups, which indicates that different control measures targeted at particular age groups should be implemented.

**Electronic supplementary material:**

The online version of this article (doi:10.1186/s40249-015-0058-9) contains supplementary material, which is available to authorized users.

## Multilingual abstracts

Please see Additional file 1 for translations of the abstract into the six official working languages of the United Nations.

## Background

*Enterobius vermicularis* infection causes enterobiasis, which is among the most prevalent parasitic diseases in children. The infection is prevalent throughout the world, including in developed countries [1–7], and it is estimated that 4–28 % of children are infected globally [8]. However, less attention has been paid to *E. vermicularis* infection because the symptoms of enterobiasis are seemingly not very severe. It is usually endemic in overcrowded conditions, such as kindergartens and primary schools, due to the easy transmission from infected to uninfected children.

Some people with *E. vermicularis* infection are asymptomatic, while others, especially children, may present with perianal pruritus, restlessness, loss of appetite, insomnia, and irritability [9–11]. Particularly, mental development in infected children lags behind their peers due to prolonged and heavy infection [12]. It has also been reported that in some rare cases, *E. vermicularis* may penetrate into kidneys and fallopian tubes, which causes ectopic enterobiasis and leads to severe health disorders and even death [13, 14].

According to the 2004 national parasitic survey, an average prevalence of *E. vermicularis* infection in children reached 10.28 % in China, with the highest prevalence rates in Hainan (42.64 %), Gansu (33.27 %), and Guangdong (30.38 %) [15]. Due to the recent rapid economic development and improvements in hygiene in Guangdong, the prevalence of three species of soil-transmitted helminthiases (ascariasis, trichuriasis, and hookworm disease) have decreased to a low level, but the prevalence of *E. vermicularis* infection is still high [16, 17]. Although many studies have been done to explore the risk factors for *E. vermicularis* infection [11, 18], related research in China is rare. However, these risk factors need to be identified in order to improve control activities. We carried out a cross-sectional survey to understand the current situation and risk factors for *E. vermicularis* infection in Gaozhou city, Guangdong province, southern China.

## Methods

### Study design

This study was conducted in Gaozhou city, Guangdong province, southern China, which has a population of near 1.3 million. Five schools were willing to participate in the survey, with two schools located in urban and three in rural areas. Children born between 1999 and 2009 were enrolled. The adhesive cellophane-tape perianal swab method was applied to detect the eggs of *E. vermicularis*. A structured questionnaire was distributed to the guardian(s) of each child in order to explore the risk factors for *E. vermicularis* infection.

### Detection of *E. vermicularis* infection

The sticky side of the transparent cellophane tape was stuck to the child’s anus between 08:00 and 09:00 in the morning. Then, the tape was removed and attached to a glass slide. Collected samples were transported to the Gaozhou Center for Disease Control and Prevention and examined under light microscopy. To increase the sensitivity, the same detection procedure was performed on three continuous days.

### Questionnaire survey

The questionnaire included questions pertaining to the basic demography of the child and her/his parents, personal hygiene and clinical symptoms of the child, as well as the family’s socioeconomic status (see Additional file 2). The basic demography section asked such things as father’s education and vocation, mother’s education and vocation, annual income of household, parents’ knowledge about controlling parasitic diseases, and so on. The children’s personal hygiene habits section wanted to find out about certain behaviors, including washing hands before dinner, washing hands after toilet use, sucking fingers, biting pencils (toys), residual dirt in fingernails, playing on the ground, washing hands after games, and playing with soil. Information about taking anthelmintics in past six months was also collected. Four related symptoms of the *E. vermicularis* infection, namely bruxism, night terrors, enuresis, and scratching the anus, were documented.

### Data analysis

Only when 90 % of each questionnaire was completed, could the data be included for analysis. Data were analyzed using the SPSS software (Version 20.0, IBM Corp., Armonk, New York). Firstly, a univariate logistic regression analysis was performed for each potential risk factor. Secondly, those variables with a *p*-value less than 0.1 were brought into the multivariate logistic regression model. Odds ratios (ORs) and 95 % confidence intervals (95 % CIs) were determined. Due to the significant difference of prevalence in ages, the children were divided into a younger age group (preschool children, under seven years of age) and an older age group (primary school children, aged seven years or above). Corresponding analyses were run again for each age group.

### Ethical considerations

The study was approved by the Ethical Review Committee of the National Institute of Parasitic Diseases, Chinese Center for Disease Control and Prevention (reference no. 2011–006). The study’s purpose, procedure, and potential risks and benefits were explained to the director and teachers of each school. It was the teachers’ responsibility to explain the study to the children’s guardians, after which a written consent was obtained from the guardian of each child. After the survey, the results were fed back to the guardians.

## Results

### Epidemiological status

A total of 825 children participated in the survey, 23 of which were excluded because over 10 % of data pertaining to them was unfounded. The overall prevalence of *E. vermicularis* infection was 54.86 % (440/802, 95 % CI: 51.42–58.31 %). The prevalence ranged from 45.96 % (74/161, 95 % CI: 38.26–53.66 %) to 68.13 % (109/160, 95 % CI: 60.90–75.35 %) in the five schools (χ^2^ = 19.212, *p* = 0.001) (see Table 1).

**Table 1:** The prevalence of Enterobius vermicularis infection in the study participants, by schools

Schools	No. of examined	No. of infected	Prevalence (%)
Changpo	172	96	55.81
Caojiang	158	88	55.70
Shankou	160	109	68.13
Hongfen	151	73	48.34
Boyu	161	74	45.96
Total	802	440	54.86

The prevalence in children from urban areas was 51.05 % (170/333, 95 % CI: 45.68–56.42 %), while it was 57.57 % (270/469, 95 % CI: 53.10–62.04 %) in children from rural areas. However, the difference was not statistically significant (χ^2^ = 3.341, *p* = 0.068).

**Fig. 1:**
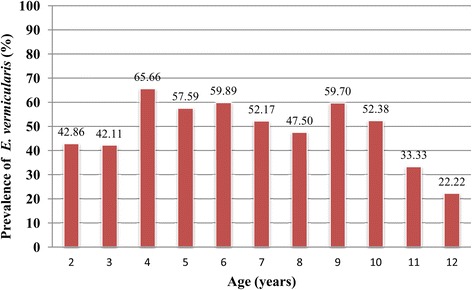
The prevalence of *Enterobius vermicularis* infection, by age groups

Of the 802 children, 480 were boys and 322 were girls. The prevalence in boys and girls was 55.63 % (267/480, 95 % CI: 51.18–60.07 %) and 53.73 % (173/322, 95 % CI: 48.28–59.17 %), respectively, which points to no significant difference (χ^2^ = 0.280, *p* = 0.596). A significant difference was observed, however, among the different age groups, with prevalence peaking in children aged four (χ^2^ = 27.190, *p* = 0.002; see Fig. 1).

### Risk factors for *E. vermicularis* infection

The results of the associations between each variable and *E. vermicularis* infection are shown in Table 2. Variables such as age, mother’s education, and washing hands before dinner were found to be significant risk factors. The younger children (two to six years of age) were more likely to be infected than older children (seven to twelve years of age) (OR = 1.63, 95 % CI: 1.20–2.20). Children who had mothers with low education levels had an OR of 1.50 times (95 % CI: 1.09–2.07) higher of being infected with *E. vermicularis*, while those who did not wash their hands before dinner had an OR of 1.52 (95 % CI: 1.02–2.28) higher.

**Table 2:** Results of logistical regression analyses of risk factors and Enterobius vermicularis infection in the study participants

Variables	No. of positive (%)	OR (95 %CI) ^a^	OR (95 %CI) ^b^	Variables	No. of positive (%)	OR (95 %CI) ^a^	OR (95 %CI) ^b^
Age group (years)				Washing hands before dinner			
7 to 12 (n = 294)	145 (49.32)	1.00	1.00	Yes (n = 671)	359 (53.50)	1.00	1.00
2 to 6 (n = 508)	295 (58.07)	1.42 (1.07–1.90) ^c^	1.63 (1.20–2.20)*	No (n = 131)	81 (61.83)	1.41 (0.96–2.07) ^c^	1.52(1.02–2.28)*
Sex				Washing hands after toilet use			
Boys (n = 480)	267 (55.63)	1.00	–	Yes (n = 584)	321 (54.97)	1.00	–
Girls (n = 322)	173 (53.73)	0.93 (0.70–1.23)		No (n = 214)	115 (53.74)	0.95 (0.70–1.30)	
Father’s education				Sucking fingers			
Middle school and above (n = 297)	153 (51.52)	1.00	–	Yes (n = 145)	79 (54.48)	1.00	–
Primary school and below (n = 505)	287 (56.83)	1.24 (0.93–1.65)		No (n = 656)	361 (55.03)	1.02 (0.71–1.47)	
Father’s floating in and out of jobs^d^				Biting pencils (or toys)			
No (n = 440)	232 (52.73)	1.00	–	No (n = 640)	343 (53.59)	1.00	–
Yes (n = 362)	208 (57.46)	1.21 (0.92–1.60)		Yes (n = 161)	97 (60.25)	1.31 (0.92–1.87)	
Mother’s education				Residual dirt in fingernails			
Middle school and above (n = 215)	103 (47.91)	1.00	1.00	Yes (n = 347)	194 (55.91)	1.00	–
Primary school and below (n = 587)	337 (57.41)	1.47 (1.07–2.01) ^c^	1.501 (1.09–2.07)*	No (n = 454)	246 (54.19)	0.93 (0.70–1.24)	
Mother’s floating in and out of jobs^d^				Playing on the ground			
No (n = 545)	288 (52.84)	1.00	–	No (n = 457)	250 (54.70)	1.00	–
Yes (n = 257)	152 (59.14)	1.29 (0.96–1.74) ^c^		Yes (n = 345)	190 (55.07)	1.02 (0.77–1.34)	
Annual income of household				Washing hands after playing/games			
>15,000 RMB (n = 237)	131 (55.27)	1.00	–	Yes (n = 451)	253 (56.10)	1.00	–
≤15,000 RMB (n = 565)	309 (54.69)	0.98 (0.72–1.33)		No (n = 348)	186 (53.45)	0.90 (0.68–1.19)	
Brought up by parents				Playing with/in soil			
Yes (n = 406)	215 (52.96)	1.00	-	Yes (n = 430)	241 (56.05)	1.00	–
No (n = 396)	225 (56.82)	1.170 (0.89–1.54)		No (n = 372)	199 (53.49)	0.90 (0.68–1.19)	
Parents‘ knowledge about controlling parasitic diseases				Taking anthelmintics in past six months			
Yes (n = 535)	296 (55.33)	1.00	-	Yes (n = 256)	132 (51.56)	1.00	–
No (n = 262)	141 (53.82)	0.94 (0.70–1.27)		No (n = 534)	300 (56.18)	1.20 (0.89–1.62)	

*OR* odds ratio, *95 %CI* 95 % confidence interval

* *p* < 0.05

^a^ Result in univariate logistic regression analysis. ^b^ Result in multivariate logistic regression analysis. ^c^ Variables entering the multivariate logistic regression analysis (*p* < 0.1 in univariate logistic regression analysis). ^d^ This signifies people who were previously employed in agriculture that leave their hometowns for another developed city and take a temporary job there to earn money

After stratification by age group, it was found that risk factors associated with *E. vermicularis* infection were different between younger and older children (see Table 3). In the younger group, children with fathers who had a primary level of education or below and who bit pencils (or toys) had a higher risk of *E. vermicularis* infection. Compared to the children whose fathers had higher education levels, children whose fathers had low education levels were 1.73 times (95 % CI: 1.20–2.50) more likely to be infected with *E. vermicularis*, and children who bit pencils (or toys) were 1.61 times (95 % CI: 1.01–2.58) more likely to be infected than children who did not. In the older age group, not washing hands before dinner and playing on the ground were the major risk factors. Compared to the children who washed their hands before dinner, children who didn’t had an OR of 1.97 (95 % CI: 1.16–3.35) times higher of being infected with *E. vermicularis*. If children played on the ground, the risk of contracting *E. vermicularis* infection increased by 1.65 (95 % CI: 1.02–2.66) times.

**Table 3:** Results of multivariate logistic regression analysis of risk factors and Enterobius vermicularis infection, by age groups

Age group	Variables	No. of examined	No. of positive (%)	OR (95 %CI) ^a^
2–6 years	Father’s education			
	Middle school and above	216	109 (50.46)	1.00
	Primary school and below	292	186 (63.70)	1.73 (1.20–2.50)*
	Biting pencils (or toys)			
	No	405	227 (56.05)	1.00
	Yes	102	68 (66.67)	1.61 (1.01–2.58)*
7–12 years	Washing hands before dinner			
	Yes	212	96 (45.28)	1.00
	No	82	49 (59.76)	1.97 (1.16–3.35)*
	Playing on the ground			
	No	126	55 (43.65)	1.00
	Yes	168	90 (53.57)	1.65 (1.02–2.66)*

*OR* odds ratio, *95 %CI* 95 % confidence interval

*p < 0.05

^a^ Result in multivariate logistic regression analysis

### Clinical symptoms

Four related symptoms of the infection, namely bruxism, night terrors, enuresis, and scratching the anus, were found in the study participants. However, the symptoms were recorded both in children with and without *E. vermicularis* infection and no significant differences were observed.

## Discussion

This study found that the prevalence of *E. vermicularis* infection in children was 54.86 % in Gaozhou, which is relatively high for China [19]. It is higher than the average level of *E. vermicularis* infection observed in Guangdong in 2004 [15] and it is also higher than the rate of soil-transmitted helminthiases in Gaozhou [16, 20]. Thus, it can be concluded that *E. vermicularis* infection is still an important parasitic disease in Gaozhou. Significant statistical difference was observed in the age variable, but not in the sex variable. The prevalence of infection was higher in the younger age group, which is consistent with the fact that younger children might be less self-aware and might not be able to self-manage. The prevalence was not significantly significant among the sexes, however, which is in line with the similarities in behavior in children of different sexes in the same environment.

In this study, several risk factors were found to be associated with a high prevalence of *E. vermicularis* infection. It was observed that *E. vermicularis* infection in children is related to not washing hands before dinner; children who washed their hands before dinner were at a significantly lower risk of contracting *E. vermicularis* infection than those who did not. This was similar to a finding in a previous report [6]. Furthermore, parents’ education levels also played an important role. Children whose mothers had low education levels had a higher risk of infection and only about a quarter of the children had mothers who had higher education levels. A study in Korea showed that parents’ knowledge about enterobiasis is correlated with the incidence of the disease in their children [11]. Combined with our findings, it can be suggested that children’s education at home needs to be strengthened in order to control enterobiasis. In addition, many children’s parents leave their hometowns to work and earn money in bigger cities (see data in Table 2: father/mother’s floating in and out of jobs), and thus some children are taken care of by their grandparents or others. Although no significant difference was found for this factor, it should be kept in mind and studied further.

It is interesting but not inconceivable that risk factors for *E. vermicularis* infection are different among the different age groups. For example, our study found that biting pencils (or toys) contributed to a higher risk factor in the younger age group, and not washing hands before dinner and playing on the ground contributed to a higher risk factor among the older age group. Taking into consideration children’s behavior at different ages, this makes sense. Biting things is more prevalent in younger children, who wouldn’t necessarily be allowed to play on the ground by themselves and whose hands would frequently be cleaned by their guardians. In contrast, older children would be allowed to play independently and thus come into contact with contaminated environments more frequently. As well as that, their parents and teachers are not as likely to help them clean their hands. We can assume that the risk of infection increases due to obvious behaviors in children, but these are easily overlooked. Thus, intervention measures should be targeted specifically to the different age groups in order to control enterobiasis. For example, parents and teachers should not only ask older children to adhere to personal hygiene practices, but also strengthen and increase supervision. For younger children, guardians should pay closer attention to cleaning pencils and toys, and prohibit their children to bite things.

Although our study has found some new and important risk factors, these alone don’t completely contribute to the high prevalence of *E. vermicularis* infection in Gaozhou. It is well known that anthelmintic therapy is the most effective approach to control *E. vermicularis* infection [21–23]. The ideal therapeutic strategy is massive chemotherapy and repeated medication [24, 25]. However, this study found that no mass-scale chemotherapy has been carried out in any school in the past year (data were collected from the director of each school). Approximately two-thirds of the children have never taken drugs for *E. vermicularis* infection in the past six months. It is therefore reasonable to conclude that this high prevalence is partially attributable to this factor. Re-infection is another important contributor. The lifecycle of *E. vermicularis* is very simple and it only takes two to four weeks for the eggs to develop into an adult worm [10]. The eggs of *E. vermicularis* can contaminate school materials, toys, door handles, desks, chairs, and even dust [26]. Children are susceptible to *E. vermicularis* infection through intimate contact with the contaminated environment and infected children [25, 27]. Thus, a high prevalence still presents in those taking anthelmintics because of rapid re-infection.

This study had some limitations and it is recommended that these are avoided in future studies. Firstly, as a cross-sectional survey, the actual relationship between cause and effect could not be ascertained [28], although the observed risk factors highlight risk of exposure, which benefits the control. Secondly, as *E. vermicularis* infection is an important parasitic disease, family aggregation should be explored, which was not done so in this study. Exploring this in future studies can further guide control measures.

## Conclusion

*Enterobius vermicularis* infection is still an important health problem in children in Gaozhou and targeted interventions are imminently needed. These interventions should integrate different measures. Firstly, massive or selective chemotherapy is important in order to decrease high prevalence rates and control the infection source. Secondly, health education should be carried out targeting children, parents, other guardians, and teachers. The difference in risk factors among different age groups urges the adoption of interventions targeted at specific age groups. Taking into consideration the high re-infection rates in collective environments such as kindergartens and primary schools, environmental hygiene should also be emphasized.

## Additional files

Additional file 1: **Multilingual abstracts in the six official working languages of the United Nations.**

Additional file 2: **Content and options of the questionnaire.**
