# Supplementary material for: Risk factors for Enterobius vermicularis infection in children in Gaozhou, Guangdong, China
Source: Infect Dis Poverty. 2015 Jun 2;4:28. doi: 10.1186/s40249-015-0058-9 (PMC4451960; doi:10.1186/s40249-015-0058-9)
Supplement: Supplementary file 1 — Multilingual abstracts in the six official working languages of the United Nations. [file 40249_2015_58_MOESM1_ESM.pdf]

Translation of the abstract into the six official working languages of the United Nations

عوامل الخطورة للإصابة بعدوى السُّرْمِيَّةُ الدُّودِيَّة لدى الأطفال في غاوجو، غوانغدونغ، الصين

هونغ مي لي، تشانغ هاي جو، جي شي لي، جو هوي دنغ، كاي ون روان، كي منغ جان، تنغ جن جو، لونغ كي كزو، و ينغ دان تشين

الموجز:

الخلفية: تعتبر عدوى السُّرْمِيَّةُ الدُّودِيَّة *Enterobius vermicularis* مرضا معويا طفيليا منتشرا بين الأطفال. في هذه الدراسة، تحرّينا الحالة الوبائية وعوامل الخطورة لعدوى السُّرْمِيَّةُ الدُّودِيَّة لدى الأطفال في جنوب الصين.

الطرق: أجري استقصاء مقطعي في مدينة غاوجو، في مقاطعة غوانغدونغ، الصين، في ديسمبر 2011. شارك أطفال تتراوح أعمارهم بين 2 – 12 سنة من خمس مدارس في هذه الدراسة. طبقت طريقة شريط السلوفان اللاصق حول الشرج لتحري عدوى السُّرْمِيَّةُ الدُّودِيَّة، في حين أرسل استبيان إلى ولي أمر (أولياء أمر) كل طالب لجمع البيانات الديمغرافية والاجتماعية الاقتصادية، إضافة إلى السلوكيات الصحية الخاصة بكل طفل.

النتائج: بين 802 طفلا خضعوا للاستقصاء، كان 440 منهم مصابون بعدوى السُّرْمِيَّةُ الدُّودِيَّة، بمعدل انتشار بلغ 54.86%، بمجال يتراوح بين 45.96% و68.13%. وقد وجد أن العمر كان عاملا ذا جدوى إحصائية، في حين لم يكن الجنس ذا جدوى إحصائية. وقد وجد أن المستوى التعليمي (المنخفض) للأم وعدم غسل اليدين قبل وجبة العشاء كانا من عوامل الخطورة الرئيسية لدى جميع الأطفال (802). بعد التصنيف حسب العمر، كان مستوى التعليم لدى الأب (ابتدائي أو أقل) وقضم أقلام الرصاص (أو الدمى) من عوامل الخطورة المميزة لدى الأطفال الأصغر سنا (508)، بينما كان عدم غسل اليدين قبل وجبة العشاء واللعب على الأرض من عوامل الخطورة الهامة لدى الأطفال الأكبر سنا (294).

الخلاصة: تظهر هذه الدراسة انتشار عدوى السُّرْمِيَّةُ الدُّودِيَّة بين الأطفال في غاوجو وتكشف عن عوامل الخطورة الكامنة وراءها. والأكثر أهمية، أنها تكشف أن عوامل الخطورة تختلف بين المجموعات العمرية المختلفة، مما يشير إلى وجوب تطبيق إجراءات سيطرة مختلفة تستهدف المجموعات العمرية المختلفة.

Translated from English version into Arabic by Lina SM, through

TRANSLATORS WITHOUT BORDERS

中国广东省高州市儿童蛲虫感染危险因素的研究

李红梅，周长海，李志式，邓卓辉，阮彩文，张启明，诸廷俊，许隆祺，陈颖丹

摘要：

引言：蛲虫是一种主要流行于儿童的肠道寄生虫。本调查旨在研究中国南部地区儿童蛲虫感染的流行病学特征和危险因素。

方法：2011 年 12 月，在中国广东省高州市开展横断面调查。调查共纳入 5 所学校，调查对象为 2 岁-12 岁的儿童。采用透明胶纸肛拭法检测蛲虫卵，同时通过问卷调查收集儿童所在家庭的人口学、社会经济学以及儿童卫生行为等资料。采用 Logistic 单因素和多因素回归方法分析相关影响因素。

结果：802 名儿童中，440 人感染蛲虫，平均感染率为 54.86%。5 所学校中最低为 45.96%，最高为 68.13%。年龄分布有统计学差异，但性别分布无差异。Logistic 多因素回归模型表明母亲文化程度低和饭前不洗手是蛲虫感染的主要危险因素。对不同年龄组儿童分层进行分析，表明父亲文化程度（初中及以下）和咬铅笔（或玩具）是低年龄组儿童（508 人）的主要危险因素；而饭前不洗手和在地上玩耍是高年龄组儿童（294 人）的主要危险因素。

结论：本研究阐明了高州市儿童蛲虫感染现状，并揭示了相关的危险因素。尤其是，本研究发现不同年龄组儿童感染蛲虫的危险因素是有差别的，这也意味着在制定控制措施时应充分考虑到不同年龄组儿童的特点。

Translated from English version into Chinese by Li Hong-mei

Facteurs de risques d'une infection due à l'*Enterobius vermicularis* chez l'enfant à Gaozhou, Guangdong, Chine

Hong-Mei Li, Chang-Hai Zhou, Zhi-Shi Li, Zhuo-Hui Deng, Cai-Wen Ruan, Qi-Ming Zhang, Ting-Jun Zhu, Long-Qi Xu, and Ying-Dan Chen

RESUME

Contexte: l'infection due à l'*Enterobius vermicularis* est une maladie intestinale parasitaire prédominante chez l'enfant. Dans cette étude, nous avons exploré le statut épidémiologique et les facteurs de risques de l'infection due à l'*Enterobius vermicularis* chez l'enfant en Chine.

Méthodes: une étude transversale a été réalisée dans la ville de Gaozhou, province de Guangdong, en Chine, en Décembre 2011. Des enfants de cinq écoles, âgés de 2 à 12 ans, ont participé à cette étude. La méthode du raclage péri-anal avec du ruban adhésif de cellophane a été appliquée pour détecter l'infection due à l'*E. vermicularis,* et un questionnaire a été envoyé aux responsables de l'enfant afin de recueillir des données démographiques et socio-économiques, ainsi que les habitudes d'hygiène de chaque enfant.

Résultats: sur les 802 enfants examinés, 440 étaient infectés par l'*E. vermicularis,* avec une prédominance moyenne de 54,86%, et un éventail de 45,96% à 68,13%. La variable de l'âge s'est avérée être statistiquement significative, contrairement à la variable du sexe. Il a été constaté que le (bas) niveau d'éducation de la mère et le non-lavage des mains avant les repas étaient des facteurs majeurs de risques chez tous les enfants (802). Après la stratification par âge, le niveau d'éducation du père (primaire ou inférieur) et le fait de mordiller des crayons (ou des jouets) étaient des facteurs significatifs de risques chez les plus jeunes enfants (508), alors que le non-lavage des mains avant les repas et le fait de jouer sur le sol étaient des facteurs importants de risques chez les enfants plus âgés (294).

Conclusion: cette étude démontre la prédominance de l'infection due à l'*E. vermicularis* chez les enfants de Gaozhou, et révèle des facteurs de risques sous-jacents. De façon plus importante, cela révèle que les facteurs de risques diffèrent selon les différents groupes d'âge, ce qui indique que des mesures de contrôle différentes ciblées sur des groupes d'âge particuliers devraient être mise en œuvre.

Translated from English version into French by Ode Laforge, through

TRANSLATORS
WITHOUT BORDERS

Факторы риска заражения острицами *Enterobius vermicularis* у детей в Гаожу, провинция Гуандун, Китай

Хонг-Мей Ли, Чанг-Хай Жоу, Жи-Ши Ли, Жуо-Хуи Денг, Кай-Вен Руан, Кви-Минг Жанг, Тинг-Юн Жу, Лонг-Кви Ксу и Йинг-Дан Чен

РЕФЕРАТ:

История вопроса: Энтеробиоз (заражение острицами *Enterobius vermicularis)* – это наиболее распространенное кишечное паразитарное заболевание у детей. В данном исследовании нами изучены эпидемиологический статус и факторы риска заражения острицами *E. vermicularis* у детей в южном Китае.

Методы: В декабре 2011 года было проведено перекрёстное обследование в городе Гаожу провинции Гуандун Китая. В обследовании участвовали дети в возрасте от 2 до 12 лет из пяти школ. Для обнаружения энтеробиоза использовался метод перианальной тампонной пробы с помощью липкой ленты, а родителям/опекунам каждого ребенка были разосланы вопросники для сбора демографических и социально-экономических данных, а также информации о гигиенических навыках каждого ребенка.

Результаты: Из 802 обследованных детей 440 были заражены острицами *E. vermicularis*, со средней пораженностью 54.86% и диапазоном от 45.96% до 68.13%. Было установлено, что возраст обследуемых является статистически значимым показателем, в то время как пол не имеет статистической значимости. Установлено, что основными факторами риска у всех детей (802) являлись образовательный уровень матери (низкий) и отсутствие привычки мыть руки перед едой. После стратификации выборки по возрасту установлено, что существенными факторами риска для младших детей (508) являются образовательный уровень отца (начальное образование или ниже) и привычка покусывать карандаши и игрушки, а для более старших детей большую значимость имеют отсутствие привычки мыть руки перед едой и игры на земле (294).

Выводы: Данное исследование демонстрирует пораженность детей острицами *E. vermicularis* в Гаожу и раскрывает основные факторы риска. Наиболее важным результатом исследования является выявление того факта, что у разных возрастных групп присутствуют специфические факторы риска, что означает необходимость применения различных мер контроля, рассчитанных на конкретные возрастные группы.

Translated from English version into Russian by Alena Hrybouskaya, through

TRANSLATORS
WITHOUT BORDERS

Factores de riesgo de la infección por *Enterobius vermicularis* en niños de Gaozhou, Guangdong, China

Hong-Mei Li, Chang-Hai Zhou, Zhi-Shi Li, Zhuo-Hui Deng, Cai-Wen Ruan, Qi-Ming Zhang, Ting-Jun Zhu, Long-Qi Xu, y Ying-Dan Chen

RESUMEN:

Antecedentes: La infección por *Enterobius vermicularis* es una enfermedad parasitaria intestinal que predomina en niños. En el presente estudio, exploramos el estado epidemiológico y los factores de riesgo para la infección por *E. vermicularis* en niños en el sur de China

Métodos: Se realizó una encuesta croseccional en la ciudad de Gaozhou, en la provincia de Guangdong, China, en diciembre de 2011. En el estudio participaron niños de cinco escuelas de entre 2 y 12 años de edad. Se utilizó la técnica de recolección perianal con cinta de celofán adhesiva para detectar la infección por *E. vermicularis,* y al mismo tiempo se envió un cuestionario a los tutores de los niños para recolectar información demográfica y socioeconómica, así como también comportamientos higiénicos de cada niño.

Resultados: De los 802 niños encuestados, 440 estaban infectados por *E. vermicularis*, con una prevalencia promedio de 54.86% y un rango entre 45.96% y 68.13%. Se encontró que la edad como variable era estadísticamente significativa, y no así el sexo. Se encontró que los factores de riesgo más importantes en todos los niños eran el nivel de educación de la madre (bajo) y el no lavarse las manos antes de comer (802). Luego de la estratificación por edad, el nivel de educación del padre (primaria o más bajo) y el morder lápices (o juguetes), eran los factores de riesgo significativos en los niños más pequeños (508), mientras que el no lavarse las manos antes de comer y jugar en el suelo eran los factores de riesgo importantes en los niños mayores (294).

Conclusión: El presente estudio demuestra la prevalencia de la infección por *E. vermicularis* en niños en Gaozhou y revela los factores de riesgo subyacentes. Lo que es más importante, revela que los factores de riesgo difieren en los distintos grupos de edades, lo que indica que deberían implementarse diferentes medidas de control dirigidas a cada grupo de edad.

Translated from English version into Spanish by Maria Alejandra Aguada, through

TRANSLATORS
WITHOUT BORDERS
