# Supplementary material for: Risk factors for Enterobius vermicularis infection in children in Gaozhou, Guangdong, China
Source: Infect Dis Poverty. 2015 Jun 2;4:28. doi: 10.1186/s40249-015-0058-9 (PMC4451960; doi:10.1186/s40249-015-0058-9)
Supplement: Supplementary file 2 — Content and options of the questionnaire. [file 40249_2015_58_MOESM2_ESM.docx]

**Content and options of the questionnaire**

1. **Basic information**
2. Child’s name: Chinese name
3. Child’s gender: boy=1; girl=2
4. Child’s age: years old
5. **Child’s family information**
6. Father’s education: illiteracy=1; primary school=2; middle school=3; senior high school=4; junior college=5; university and above=6
7. Father’s vocation: farmer=1; worker=2; floating out-for-jop=3; businessman=4; public functionary=5; others=6
8. Mother’s education: illiteracy=1; primary school=2; middle school=3; senior high school=4; junior college=5; university and above=6
9. Mother’s vocation: farmer=1; worker=2; floating out-for-jop=3; businessman=4; public functionary=5; others=6
10. Annual income in household: < 5 000RMB=1; 5 000-15 000RMB=2; > 15 000RMB=3
11. Child brought up by whom: grandparents=1; maternal grandparents=2; parents=3; baby sitter=4; others=5
12. Parents’ knowledge on controlling parasitic diseases: Yes=1; No=2
13. Taking anthelmintics in recent 6 months: Yes=1; No=2
14. **Personal hygiene habits**
15. Washing hands before dinners: Yes=1; No=2
16. Washing hands after toilets: Yes=1; No=2
17. Sucking fingers: Yes=1; No=2
18. Biting pencils (toys) : Yes=1; No=2
19. Dirt residual in fingernails: Yes=1; No=2
20. Playing on the ground: Yes=1; No=2
21. Washing hands after games: Yes=1; No=2
22. Playing soils: Yes=1; No=2
23. **Child’s clinical symptoms**
24. Scratching anus: Yes=1; No=2
25. Night terrors: Yes=1; No=2
26. Enuresis: Yes=1; No=2
27. Bruxism: Yes=1; No=2
